# Estrogen Signaling in Bone

**DOI:** 10.3390/app11104439

**Published:** 2021-05-13

**Authors:** Nuria Lara-Castillo

**Affiliations:** Department of Oral and Craniofacial Sciences, School of Dentistry, University of Missouri, 650 East 25th Street, Kansas City, MO 64110, USA;

**Keywords:** estrogen, estrogen receptor, bone homeostasis

## Abstract

Estrogen plays important roles in bone homeostasis throughout a person’s life, including longitudinal bone growth, bone healing, and adaptation to mechanical forces. Estrogen exerts its action by binding to its multiple receptors in the cell membrane and cytoplasm. Until now at least three estrogen receptors (ER) have been reported: ER alpha (ERα), ER beta (ERβ), and G-protein coupled estrogen receptor 1 (GPER1) also known as GP30. Recently it has been observed that estrogen crosstalk with other signaling pathways helping to understand its wide effects in bone homeostasis. Abrupt loss of estrogen production experienced by menopausal women is associated with the rapid loss of bone mass ultimately leading to osteoporosis. The detrimental results during its absence with aging and the increased life expectancy of current and future generations make it of high importance to fully understand its mechanism of action. This review article aims to update on (1) the molecular mechanism of action of estrogen in the skeletal system, (2) ERs expression in different bone cells, (3) recent reported ER mutations resulting in pathological human conditions, and (4) role of estrogen signaling during bone healing.

## 1. Introduction

Estrogen, a sex hormone derived from cholesterol, plays critical functions in both females and males. Women produce three types of estrogen throughout their lifespan: 17β estradiol (E2) starting at puberty until menopause, estriol (E3) during pregnancy, and estrone (E1) after menopause. There are two other estrogens: estetrol or E4 (produced by the fetal liver) and estrone sulfate (E1S) that mainly serves as a reservoir for the excess of estrogen (formed by the action of the enzyme estrogen sulfotransferase on E1) [1]. During the childbearing period, women produce estrogen cyclically, with circulating blood levels in the range of 20–350 pg/mL (according to the Mayo Clinic Laboratories). The main production of estrogen (~85%) occurs in the ovaries, but the suprarenal gland and the adipose tissue can produce small amounts of estrogen. After menopause, estrone is produced in ovaries in addition to several other organs. Men produce estrogen in lower quantities, with circulating levels in the range of 10–40 pg/mL (according to the Mayo Clinic Laboratories). Most of the estrogen found in men is synthesized from testosterone by the action of the aromatase enzyme (encoded by the CYP19A1gene) [2,3]. Testes and the brain produce measurable amounts of estrogen [4] while adrenal glands produce just a small amount.

In women, the main function of estrogen is to mature and maintain the reproductive system; however, the impact of estrogen does not stop there. It also influences other physiological functions, as well as psychological behaviors. Its wide effects are obvious during menopause when levels of E2 production by the ovaries sharply decline. After menopause women experience physical changes, including the decrease of bone mineral density leading to a high risk of bone fracture, increase in the incidence of cardiovascular problems, hot flashes, dry eyes, and dry vaginal mucosa, among other body changes [5,6]. The psychological changes observed are depression, anxiety, irritability, and a decrease in sex drive [7]. The role of estrogen in men’s physiology is mainly related to bone health, brain [8], and sexual function [9].

Because of the wide effects of estrogen on women’s well-being and its sharp decrease in production during menopause, the golden treatment has been to restore levels of estrogen by hormonal replacement therapy (HRT). There have been some concerns regarding this approach due to the observed increase of breast cancer, coronary heart disease, and stroke as recently reviewed [6]. However, new stratification of the data and reassessment of clinical trials, HRT now shows to be beneficial with low risks in women who start therapy at the onset of menopause [10]. This new analysis of the data gives hope for women undergoing menopause and those who due to health issues had undergone hysterectomy (removal of the reproductive system).

Due to its critical function in several tissues, it is important to fully understand the precise mechanism of how estrogen works hence new therapies can be developed to improve women’s and men’s health during aging without the side effects currently observed. This review aims to update on the role of estrogen and its mechanism of action in the bone.

## 2. Estrogen in Bone

### 2.1. Estrogen in Bone

Nowadays, it is well-recognized that estrogen is important in bone homeostasis playing a pivotal role in longitudinal bone growth in both male and female skeletons. In females, during the early stages of puberty, low levels of estrogen allow for the rapid growth of the bone, and towards the end of puberty high levels of estrogen result in the closure of the growth plate [11]. In males, it was previously thought that androgen was responsible for skeletal growth. This hypothesis changed after the reports and careful analysis of two anomalies in the male skeleton involving estrogen synthesis and signaling pathway. The first one was the clinical report of a male patient exhibiting bone growth even into adulthood. This patient carried a mutation in codon 157 in both alleles of the estrogen receptor alpha (ERα) changing a cytosine for thymine resulting in a premature stop codon. This mutation resulted in tall stature due to the lack of growth plate fusion. Despite the abnormal length of the bone and high levels of testosterone, he had low bone density (3.1 SD below the mean for age-matched normal women), resulting in osteoporosis [12]. The second finding was the identification of two male patients with similar clinical characteristics as previously described patients, but in their cases, an aromatase p450 deficiency was responsible for the same clinical outcome [13,14].

### 2.2. Mechanism of Action

**Figure 1.**
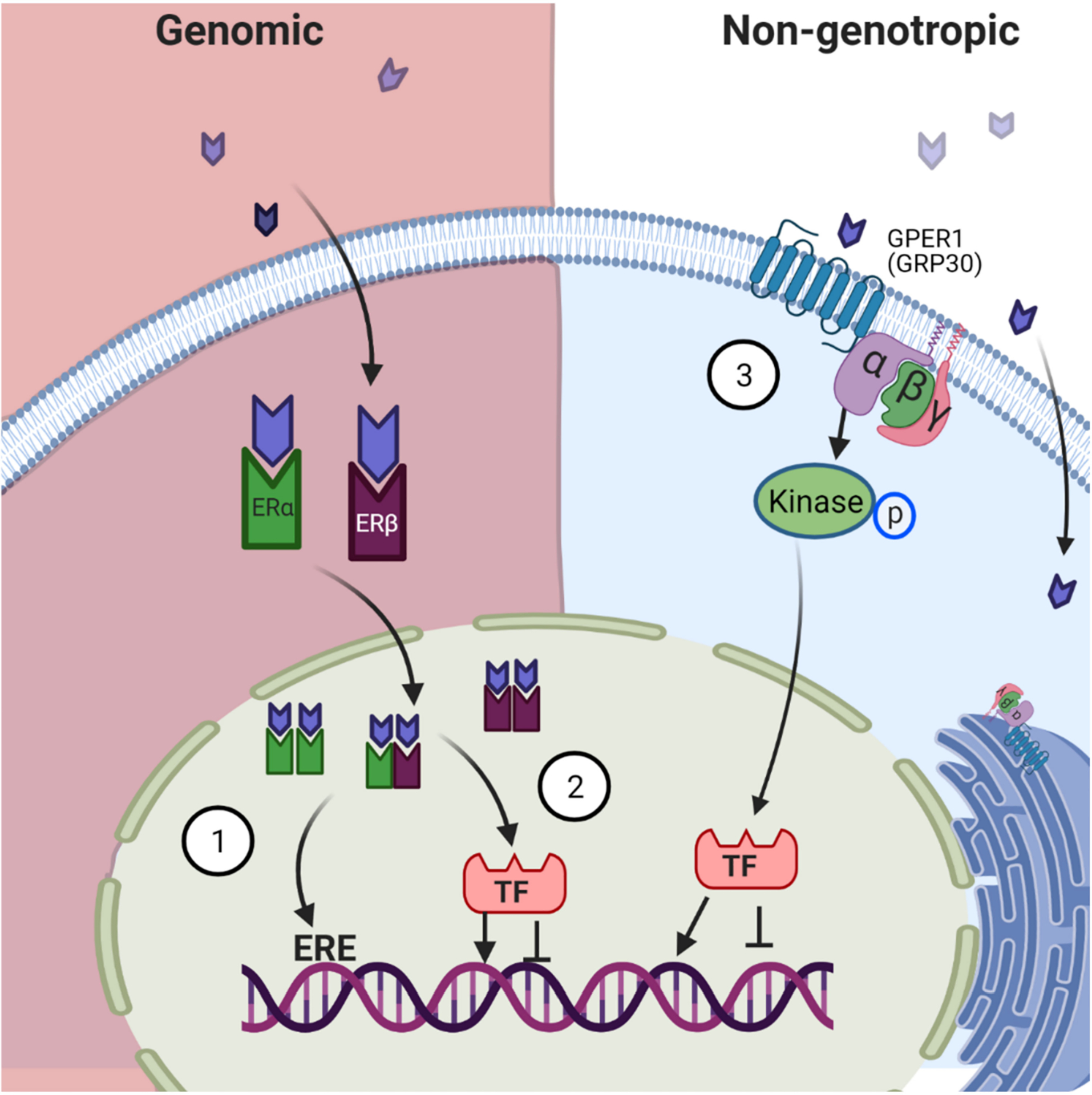
Traditional Mechanisms of Estrogen Signaling. Genomic signaling: Estrogen diffuses through the cell membrane into the cytosol of the cell where it binds to its receptors. Estrogen-Estrogen Receptor (E-ER) complex moves to the nucleus where it forms homo- and/or heterodimer complexes. This complex either (1) binds to the specific Estrogen Responsive Elements (ERE) in the DNA or (2) recruits transcription factors which then alter transcription. Nongenotropic signaling: (3) Estrogen binds to G protein-coupled estrogen receptor (in the plasma membrane) and activates downstream signaling mechanisms. These three signaling mechanisms ultimately lead to altered gene expression.

Estrogen, like other steroid hormones, exerts its action by binding to its receptor via at least three mechanisms (see Figure 1): (1) The classic genomic signaling: estrogen enters the cell and binds to its receptors α and β (ERα, ERβ) in the cytoplasmic compartment; this complex then moves to the nucleus where it forms homo- or heterodimers and binds directly to a specific DNA sequence called Estrogen Response Elements (ERE). (2) The second mechanism is ERE-independent: Estrogen-ER complexes move to the nucleus, interact with other transcription factors through protein-protein interaction, sequestering them, therefore, modifying their interaction with DNA leading to alteration in gene expression [15]. (3) The third mechanism of action is referred to as non-genotropic signaling. Here estrogen signals through a G-protein-couple receptor (GPCR) on the plasma membrane. Recently G protein-coupled estrogen receptor-1 (GPER1), also known as GPR30, was identified as a receptor for estrogen in the plasma membrane. This binding activates downstream signaling cascades that ultimately alter gene expression [16]. In addition to the traditional estrogen receptor signaling, genomic and non-genotropic just discussed, a new mechanism of action has recently been reported in other fields of studies (cancer field), epigenetic regulation by estrogen receptor. ERα regulation of histone acetylation is achieved by the recruitment of coactivators that have histone acetyltransferase activity [17,18]. ERα also interacts with methylases and demethylases leading to the regulation of targeted genes [19]. Another new mechanism of estrogen receptor signaling is the activation of the receptor in the absence of ligand stimulation. Caizzi et al. (2014) reported that unliganded ERα binds to more than 4000 chromatin sites that are specially linked to genes with developmental functions participating in the homeostasis of luminal epithelial cells [20].

## 3. Estrogen Receptors

### 3.1. Identification

Discovery and subsequent cloning of the estrogen receptor were paramount steps not only for the estrogen signaling field but also for the discovery of the nuclear receptor superfamily. In 1960, a revolutionary theory of a plausible receptor for estrogen started to take shape. By using tritium-labeled estrogen, which is highly radioactive, Jensen and colleagues paved the way for the discovery and identification of such molecule [21]. When physiological amounts of tritium-labeled estrogen (90 ng) were administered to immature female rats, the uterus and vagina (tissues that respond to estrogen treatment with dramatic growth) showed higher uptake (100–200 times higher concentration than blood) and longer retention of the radioactively labeled hormone (peak levels lasted for almost 2 h). The liver and kidneys (tissues in which substantial steroid metabolism and degradation occurs) also show high levels of radioactivity; however, the peak concentration was observed at 15 min in these organs. These results revealed two major breakthroughs: (1) at low levels in the blood, estrogen targets specific tissues such as the uterus; and (2) estradiol is not metabolized in the uterus. With the results from these experiments, Jensen and colleagues proposed that estrogen was held in the cytoplasm by a specific protein, which we now refer to as the estrogen receptor, and it is this complex that moves to the nucleus where it exerts its action.

In 1966, six years later, Toft and Gorski reported data on the isolation of a macro-molecule bound to estrogen, with the characteristic of a receptor, which was only found in the uterus, but not in the small intestine nor blood serum. The binding was antagonized by diethylstilbestrol, but not by non-estrogenic steroids, testosterone, or corticosterone [22]. Twenty years later, in 1986, in collaboration with Pierre Chambon’s group, the gene for estrogen receptor was cloned. The clone, now referred to as ERα, encoded 595 amino acid residues with a calculated molecular weight of 66 kDa [23].

For several decades after its discovery, ERα was the only estrogen receptor known to the scientific community; therefore, the discovery of a second estrogen receptor in 1996 was a surprise to the field. Kuiper et al. identified and cloned the ERβ from a rat prostate cDNA library while screening for additional androgen receptors [24]. The ERβ clone encoded 485 amino acid residues with a calculated molecular weight of 54.2 kDa. Authors reported that this novel estrogen receptor had a high homology at the DNA binding site (95%) to the already known estrogen receptor (ERα) and a high affinity for estradiol (Kd = 0.6 nM). Using in situ hybridization, they reported high expression in the prostate, in epithelial cells of the secretory alveoli, and the granulosa cells in the primary, secondary, and mature follicles of the ovary [24].

Like other nuclear receptors, the two ERs seem to have opposite effects. In a classic paper, Hall et al. (1999) eloquently demonstrated that ERβ functions as a trans-dominant inhibitor of ERα at sub-saturating hormone levels. The authors transfected HepG2 and HeLa cells with ER plasmids. They used these cell lines because they depend on exogenous ERα and ERβ to activate ERE-dependent transcription. In the presence of 100nM of 17β estradiol, ERα was able to activate gene expression regardless of ERβ expression. However, at sub-saturating hormone levels (100 pM), the ability of ERα to activate gene expression was significantly decreased as the levels of ERβ increased in the system. In this paper, they also support the hypothesis that ERβ might be binding to its cognate response element in a constitutive manner, competing with ERα for access to DNA targets [25].

In 2005, almost 10 years after ERβ discovery, two groups reported that 17β-estradiol binds to a seven trans-membrane bound estrogen receptor, G protein-coupled estrogen receptor (GPER1) or G-protein-couple receptor 30 (GPR30) located in the plasma membrane and endoplasmic reticulum. This novel receptor is responsible for the rapid non-genomic cell signaling by intracellular calcium mobilization and synthesis of intracellular 3,4,5-triphosphate in the nucleus [26,27].

### 3.2. Estrogen Receptor Structure

**Figure 2.**
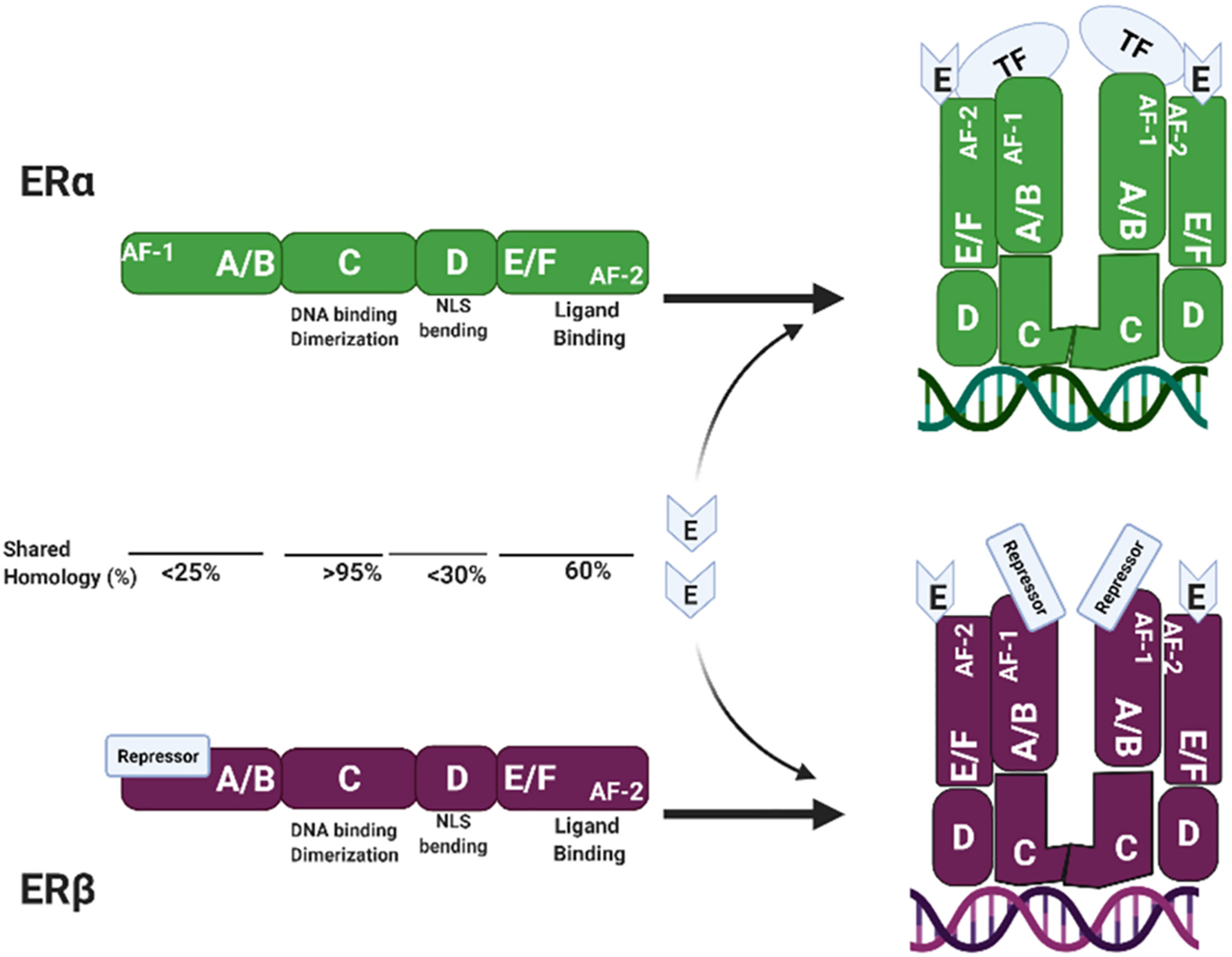
Estrogen Receptors Structure. Estrogen receptors α and β share similar characteristics. They are formed by 6 domains and have shared homology, being the DNA Binding Domain, the most highly conserved. They both have the D domain where the nuclear localization signal (NLS) is located and a hinge for the protein to fold. In the E/F domain, they have the ligand-binding domain (LBD) and the AF-2 which is ligand-dependent. They differ in the A/B domain, ERα has an activator factor (AF-1) that is ligand-independent, whereas ERβ has a repressor site. Upon binding to Estrogen there is a conformational change and a folding of the receptor at the D domain, bringing the C-terminal close to the N-terminal and in the case of ERα attracting transcription factors (TF).

ERα and ERβ are members of class I nuclear receptors. They are ligand-activated receptors and can dimerize into homo- or heterodimers. Even though ERα and ERβ are encoded by two different genes located in two different chromosomal loci, 6q25.1–25.2 and 14q23.2–q23.3, respectively, they have similar overall structure and share a high degree of amino acids homology. Börjesson et al. (2013) nicely describe the structure of the estrogen receptors. There are six domains in their structure, named A to F (see Figure 2).

The A and B domain, at the N-terminal, encodes for the ligand-independent Activation Function-1 (AF-1). The C domain encodes for the DNA-binding domain and it is also important for dimerization. The D domain contains the nuclear localization signal (NLS) and an area of bending allowing the C domain to come close to the E and F domains which contain the ligand-binding domain, along with the ligand-dependent Activation Factor-2 (AF-2). When estrogen binds to the ligand-binding domain, there is a conformational change that brings the AF-2 domain, in the C-terminal, close to helices 3, 4, and 5 located in the N-terminal of the receptor. This new conformation allows for the recruitment of cofactors that will ultimately regulate gene expression [28]. The main difference between the two receptors is in the amino-terminal. In the ERα, this area encodes for the AF-1, that when it comes into proximity with the AF-2 sequence, it recruits transcriptional factors leading to activation of targeted genes. In the ERβ, this area may encode for a repressor domain that exerts the antagonistic effects seen in the different systems [25]. The ligand-binding domain is 60% conserved between the two estrogen receptors, but the NH2-domain, including the transcriptional activation domain AF-1, is less than 25% conserved.

### 3.3. Differential Expression of Estrogen Receptors in Bone

All estrogen receptors are differently expressed in bone cells. In 1998, Kusec et al. reported the expression of mRNA ERα in the chondrocytes, active osteoblasts, and lining cells on trabecular surfaces of both human (9–15 years of age) and rabbit bones. They were unable to detect mRNA expression in osteocytes or osteoclasts in either species [29]. In 1999, Kennedy et al. reported the immunolocalization of ERα in the growth plates of rabbits, but not in rats, during sexual maturity [30]. In the same year, Gruber et al. reported the expression of ERα and ERβ in total bone marrow, as well as in primary murine marrow stromal cell cultures, at the mRNA, as well as at the protein level [31]. Orefo et al. (1999) reported the expression of ERα in the human pre-osteoclast cell line, TCG 51, but its expression was not observed in the more mature, bone resorptive TGC23 cell line. Neither could they detect expression of ERα in samples obtained from patients with conditions known to increase osteoclast numbers such as hyperparathyroidism, CGTB, and Pagetic bone [32]. Arts et al. (1997) showed differential expression of both ERs mRNA during differentiation of human fetal osteoblast-like cell line (SV-FHO). They reported that while ERβ mRNA levels increased during osteoblast differentiation, levels of ERα were kept constant after day 10 of differentiation (the moment of maximal expression of alkaline phosphatase) [33].

In the early years, after ERβ discovery and cloning, several groups reported expression of ERβ in the bone of several species from rats [34] to humans [35]. More specifically, they reported that in areas of active bone formation or bone remodeling, ERβ is highly expressed in the nuclei of osteoclasts, and less expressed in cells of the osteoblastic lineage. Cells in the hypertrophic cartilage are negative for ERβ, but those close to endochondral osteogenesis are positive [35]. Bord et al. (2001), using human neonatal rib bone, reported that ERβ is mainly expressed in trabecular bone with little expression in cortical bone. The opposite being for ERα, which is highly expressed in cortical bone and low expressed in trabecular bone. Most osteocytes in the cortical bone, as well as osteoblasts within the osteons and on the periosteal and endosteal surfaces, showed intense ERα staining. Osteocytes within the trabecular bone express low levels of ERα, but with the expression more intense in those cells closer to newly mineralizing osteoid [36].

GPER1/GPR30 is present in the growth plate of young humans but its expression declines during puberty [37]. Its expression has been confirmed in bone marrow mesenchymal stem cells [38], hypertrophic chondrocytes [37], osteoblasts, and osteocytes [39]. Just recently, Chuang et al. (2020) reported that GPER1/GPR30 mediates bone marrow mesenchymal stem cell proliferation via the cAMP/PKA/CREB pathway [38].

The expression of estrogen receptors is regulated by the methylation state in the promoter region. Their lack of expression due to hypermethylation in the promoter region is related to cancer development in bone and metastasis of tumors in bone. ERα is normally expressed in osteoblasts, but osteosarcoma cells does not express ERα due to hypermethylation in its promoter region [40]. Re-expression of ERα in 143B osteosarcoma cells with Decitabine (a hypomethylating agent) induced osteosarcoma cell differentiation and decrease proliferation. These effects were not observed ERα knockout treated with Decitabine [41]. Leav et al. demonstrated an inverse relationship between the extent of ER-beta CpG islands (CGIs) methylation and receptor expression in normal, hyperplastic, premalignant, and malignant foci of the prostate and lymph node and bone metastases [42]. Zhao et al. later demonstrated methylation in two CGIs of the ER beta promoter region are insignificant in the normal epithelium in the prostate, reached 80–90% in grade 4/5 in prostate cancer, but declined to less than 20% in bone metastases [43].

### 3.4. Role of ERα in Bone

ERα is highly expressed in bone and most of the effect of estrogen has been attributed to activity mediated through this receptor. This was proven when ovariectomized (OVX) ERα^−/−^ female mice and orchiectomized (ORX) ERα^−/−^ male mice did not respond to exogenous estrogen [44–47]. Studying the effect of ERα in bone using a global knockout approach (ERα^−/−^) gives a general idea of what its role might be. However, this approach has proven to have the downfall that alters other hormone levels. ERα^−/−^ mice show a 10-fold increase in estradiol and a 5-fold increase in testosterone levels, as well as altered growth factors such as IGF-1 [44]. This alteration can lead to misinterpretation of the role and importance of the estrogen receptors in bone. One way to overcome this pitfall is using the LoxP/Cre system. In the last five years, two papers have reviewed the different bone-targeted conditional ER knockout mouse models. Khalid and Krum (2016) outlined the different knockout mouse models of ERα and ERβ (global and tissue-specific knockouts) [48] and Rooney and van der Meulen (2017) reviewed the different conditional ERα mice knockouts reported with a summary of their response to mechanical loading [49]. In summary, deletion of ERα in osteoblast progenitors and precursors using either Prx-1 or Osx-1 Cre models lead to the reduced cortical thickness and decrease bone mineral density (BMD) in females until 28 weeks of age. Male ERαf/f;Prx1-cre mice also had lowered femoral BMD and decreased cortical thickness up to 8 weeks of age; however, this difference was no longer presented in 18-week-old male ERαf/f;Prx1-cre mice. When ERα was deleted in osteoblast precursors using the 2.3 kb Col1a1 Cre mouse model, there was no difference between control and conditional knockout in cortical or trabecular bone in either sex [50]. Deletion of ERα in mature osteoblasts using the Osteocalcin Cre mouse model shows a decrease in both cortical and trabecular bone parameters in young female mice, 3.5-month-old, and no difference in young males. However, at 6 months of age, male knockout had lowered trabecular bone volume [51]. Deletion of ERα in osteocytes using the Dmp-1 Cre led to a decrease in trabecular bone mass in male mice and no change in cortical bone mass in either male or female [52]. These results were in part contrary to those reported by Kondoh et al. (2104) where authors observed changes in trabecular parameters in female but not male mice; and like previously reported, no changes in cortical bone [53]. Deletion of ERα in osteoclast precursors using the LysM Cre mouse model shows no changes in the cortical bone at 3 months of age. However, trabecular bone mass was decreased at 6 months of age in females. Unfortunately, males were not studied in this paper. Deletion of ERα in mature osteoclasts using the Cathepsin-k Cre mouse model showed an increase in bone resorption resulting in decreased trabecular bone in females but not males [54].

### 3.5. Role of ER Beta in Bone

Most of the knowledge obtained about ERβ function has come from global knockout rodent models. Like ERα global knockout mice, this approach has many limitations and it is important to note that ERβ global knockouts mice show expression of spliced variants present in several tissues including bone that can lead to confounding results [55–57].

Several groups have created and characterized different ERβ knockout mice: βERKOCH, βERKOKI ERβKOST, ERβKOWY, and ERβSTL-/L-. The nomenclature refers to the institute in which the mutant was developed and differs mainly on how the deletion occurs. Krege et al. generated the ERβKOCH (mice produced in the University of Carolina, Chapel Hill, NC) by disrupting exon 3, which encodes the first zinc finger of the DNA Binding Domain, by insertion of a neomycin-resistance gene (Neo) cassette in the reverse orientation into the PstI site. In this animal model, no full-length mRNA was detected; however, several transcript variants were observed in which exon 3 was consistently spliced out. Mice lacking full-size ERβ reproduced normally but had fewer and smaller litters than wild-type mice. This may be due to reduced ovarian efficiency. Older male knockout mice suffered from the bladder and prostatic epithelium hyperplasia [58]. This mutant was then transferred to the Karolinska Institute (βERKOKI) in Sweden where mutant males developed prostatic intraepithelial hyperplasia. Researchers at this institution determined these mice were not only sterile, but they also had questionable behavioral conduct, leading to support the hypothesis that ERβ is involved in the development and/or homeostasis of the brain. The female mutant mice showed a lack of ovulation [59].

In 2002, Sims and colleagues reported the generation of a second ERβ knockout mouse (ERβKOST) in Strasbourg, France. They inserted a Neo gene in the SpeI site of exon 3 in the 5′ to 3′ orientation. Like the knockout previously described, authors detected the presence of several spliced variants lacking exon 3. However, they could not confirm the epithelial hyperplasia of the bladder and prostate ducts observed in the βERKOCH mouse model. In this animal model, it was determined that global deletion of ERβ did not affect male skeleton but in females, bone resorption was decreased resulting in an increase in trabecular bone volume [57].

A third ERβKO was generated at the Wyeth-Ayerst Research/Genetic Institute (ERβKOWY), which was different from those previously described. The construct used in this animal model introduces stop codons in all three reading frames of the ERβ gene, leading to a complete knockout. In addition, they introduced a Neo cassette in the reverse orientation resulting in the truncation of exons 1 and 2 and deletion of intron 1. The authors probed for ERβ protein expression in the brain and ovaries, tissues known to have high ERβ expression, and confirmed deletion of the receptor in those tissues. No bone analysis was reported in this paper [60].

A fourth ERβ-null mouse model was reported in 2007 (ERβSTL-/L-). This is different than previously reported in that it was created using the Cre/LoxP-mediated excision. Authors claim it lacks any spliced variants downstream of exon 3; furthermore, this animal model can be used to delete ERβ in specific tissues using the Cre/LoxP system. These animals developed none of the anomalies reported in previous animal models that appear during aging (prostatic hyperplasia, defects in several tissues including brain, lungs, heart, urinary bladder, and intestine). The authors reported that ERβSTL-/L- mice of both sexes are sterile. In female ERβSTL-/L- mice, infertility comes from the lack of ovulation. Infertility in male mice was unknown to the authors when the paper was published since the gonads and internal genital organs appeared normal but probably were not functional [61]. Several groups have used this animal model since its creation. Deletion of ERβ resulted in increased mineral content in the cortical but not in the trabecular compartment and increased longitudinal growth in young animals [55]. ERβ deletion resulted in protection from age-related trabecular bone loss [56]. Using this ERβSTL-/L-, Nicks et al. (2016) specifically deleted ERβ in osteoprogenitor cells (mesenchymal precursor cells) in the appendicular skeleton using the Prx1-Cre model system (ERβPrx1CKO). The authors argued that since no regulatory effects were seen in males in previous studies, and they only studied females for this paper. They reported an increase in trabecular, but not cortical, bone mass due to an increase in trabecular number rather than trabecular thickness. They confirmed these findings by crossing the ERβSTL-/L- with the Col2.3-Cre driver which deletes the ERβ in early osteoblasts. At the molecular level, deletion of ERβ in osteoprogenitor cells down-regulated several pathways related to bone formation, including autophagy, adipogenesis, senescence, circadian rhythm, Wnt signaling, oxidative stress, and apoptosis among others [61].

### 3.6. Role of GPER1/GP30 in Bone

Effects on bone due to the global or targeted deletion of the GPER1/GP30 are still in their early stages. Only a couple of groups have studied the effects of deletion of this receptor in the musculoskeletal system. Female mice in which G protein-coupled receptor was deleted using a global knockout approach, showed reduced crown-rump length and reduced femur length. These differences were not observed in males [62]. Another study showed that male mice carrying a Gpr30 null mutation showed increased whole-body, spine, and femoral areal bone mineral density, increase trabecular bone volume, and cortical thickness [63].

### 3.7. Estrogen Receptor Mutation in Humans

The involvement of estrogen in bone homeostasis started with the clinical observations of male patients with anomalies in the estrogen synthesis and signaling pathway (described earlier in this review). More specifically, the mutation in the ESR1 (gene encoding ERα) and the CYP19A1 gene encoding the aromatase enzyme. While mutations in ESR1 are rare in primary tumors (less than 1%) recently has been reported that the prevalence of ESR1 mutations is higher in diagnosed metastatic patients (6–55%), including in patients with bone metastasis. These mutations in the ligand-binding domain (LBD) region of ER result in ligand-independent constitutively activated receptors. The most common ESR1 hotspot mutations are D538G [64], L536R, and Y537S/N/C [65–68]. Mutations in the ESR2 (gene encoding for ERβ) have been primarily involved in breast cancer development. It was not until recently that bone abnormalities involving a mutation in the ERβ were reported. In 2018, two papers were published reporting ESR2 mutations in four unrelated patients with syndromic and non-syndromic 46, XY disorder of sex development (DSD) [69,70]. 46, XY DSD patients have one X chromosome and one Y chromosome, a pattern usually found in males. At birth, it is not clear whether these patients are male or female because patients have genitalia but have an undeveloped or complete absence of female reproductive organs. Clinically these patients suffer from absent breast development, primary amenorrhea, abnormal or absent gonadal development, and delayed bone age. Several gene mutations in nuclear receptors have been linked to this disorder but they still do not account for all the cases. Here four cases of DNA sequences analysis are described. Case#1: one of the patients revealed a homozygous 3-bp deletion in exon 9, c.541_543del, which leads to in-frame deletion, p.(Asn181del), affecting a highly conserved amino acid located in the DNA Binding Domain (DBD). Two other patients showed heterozygous ERS2 missense variants: case#2 had a c.251G>T found in exon 2 leading to a missense change p.(Gly84Val). This mutation affects a highly conserved amino acid located in the N-terminal where the first activation function (AF-1) is located. Case#3: the patient had c1277T>G in exon 8 of ESR2 leading to a missense change p.(Leu426Arg) in the ligand-binding domain [70]. Case #4: the patient had a heterozygous missense mutation, a transition from A to G in the fifth codon of ESR2 that translates in a Lys314Arg, and based on molecular modeling, this mutation is predicted to impair interaction with nuclear coactivator 1 (NCoA1) [69].

### 3.8. Role of Estrogen and Its Receptors in Bone Regeneration

Fracture healing is a series of postnatal events that resemble biological processes taking place during the embryonic development of the skeleton (for review refer to [71]). This complex process occurs in three marked stages: inflammation, callus formation, and bone remodeling. Each step is characterized by a series of cellular and biochemical events.

At the site of injury, there is disruption of vascular vessels and formation of a hematoma [72]. This leads to an almost immediate increase in the expression of inflammation markers such as tumor necrosis factor (TNFα) [73], interleukins (IL) 1β, 6, 7, 11, 17A & 18 [74]. This environment promotes the movement and infiltration of inflammatory cells allowing for the recruitment of mesenchymal stem cells (MSCs) from the bone marrow [75], periosteum [76,77], muscle, and peripheral blood supply (see [78] for review). The second step is callus formation. At the inner core of the injury site, there is a remarkable depletion of oxygen due to the disrupted vasculature. It is at this location that bone regeneration occurs via endochondral bone formation. The newly recruited MSCs undergo chondrogenesis: proliferation, hypertrophy, and terminal differentiation. This step is marked by the increased expression of chondrocyte markers including Sox-9, Col II, Col X, MMP-9, MMP-13, and Ihh. The newly differentiated chondrocytes start producing vascular endothelial growth factor (VEGF) [79] to promote the formation of new blood vessels in the injured area, a process called angiogenesis. Then, chondrocytes undergo apoptosis attracting osteoclasts to the area to start the bone remodeling process. This stage is marked by an increase in osteoclasts-related genes such as M-CSF (macrophage colony-stimulating factor), RANK, RANK-L, and osteoprotegerin (OPG). At the periphery of the fracture where vasculature is still intact, bone regeneration occurs via intramembranous ossification which is the process when the bone is formed from a fibrous membrane. The late phase of fracture healing is characterized by the formation of the neocortex and its remodeling to mature lamellar bone. During this late stage, the healing process is characterized by the remodeling of the periosteal callus which is enhanced in the presence of estrogen.

Estrogen plays an important role in all aspects of bone healing. In callus formation by enhancing cartilage homeostasis, growth, and differentiation [80]. In 2010, Beil and colleagues showed that ovariectomized (OVX) mice subjected to bone fracture have less newly formed cartilage at the fracture gap (at 7 and 14-days post-fracture) as compared to sham and estrogen-treated groups. In the same paper, they reported that the estrogen-treated group had increased chondrocyte formation compared to Sham and OVX mice. Authors have noticed that there were no differences in terms of periosteal callus size, femur diameter, or BMD in the fracture area up to this point of the fracture healing process [81]. Estrogen also plays an important role in the last step of bone remodeling. The ability to remove cartilage and allow deposition of new mineralizing cells into the area is critical in the fracture healing process, and lack of estrogen impairs this process. In OVX mice, the amount of cartilage remaining in the fracture site is higher when compared to control groups of mice receiving estrogen treatment, the latest group having the highest mineralized area [81].

As described previously in this review, estrogen signals through its receptors, ERα, ERβ, and GPER1/GPR30, and it should not be surprising to expect that these receptors play an important role in the bone healing process. Recently, Wu et al. (2020) studied the levels of ERs in the bone healing process using a metaphyseal bone defect. Mice were subjected to a monocortical bone circular perforation (1.0 mm diameter) of the femur. In this bone healing model, the authors reported an increase in ERα protein expression, but not ERβ, at the injury site. Interestingly, they showed that increased expression of ERα colocalized with the high expression of mitochondria in the bone-defect area and the increased expression of *CoxI* and *CoxII* mRNA, genes important in ATP production in the mitochondria. Authors reported an increase in the expression of genes with an ERE (estrogen-responsive elements) such as *Runx2*, *alp* (alkaline phosphatase), and *ocn* (osteocalcin). In addition, levels of PECAM-1, a biomarker of angiogenesis, were increased in the bone-defect area. All these changes were blocked in mice treated with an ERα inhibitor, methylpiperidinopyrazole (MPP) at a concentration of 2 mg/kg of body weight for 14 days after bone defect surgery. Authors concluded that the estrogen-ERα signaling axis is involved in bone healing via energy production, osteoblast maturation, and angiogenesis [82].

Low-magnitude high-frequency vibration (LMHFV) has proved to be a non-invasive treatment to increase bone formation in poorly healing fractures. However, the amount of estrogen present might be of importance. Wehrle and colleagues (2015) showed that LMHFV disturbed fractured healing in aged non-OVX mice. At the molecular level, the authors found an increase in *ERβ* and *Sost* expression and a decrease in β-catenin in the callus of the vibrated animals. Conversely, LMHFV increases callus properties that could be abolished by the administration of subcutaneous estrogen slow-release pellet. At the molecular level, the authors reported an increase in ERα expression in the callus of the vibrated OVX mice with no change in the expression of ERβ [83].

Up to now, no human studies are reporting the use of estrogen-related drugs in the improvement of fracture healing. Although selective estrogen receptor modulators (SERMs) are not used as a direct treatment for osteoporosis or bone healing, there are beneficial effects observed in patients treated with this type of drug. SERMs belong to a group of drugs that are either agonists or antagonists of estrogen receptors in a tissue-specific manner. Tamoxifen and raloxifene, among others, are part of breast cancer therapy in ER+ patients. They inhibit breast cancer cell proliferation and, at the same time, they behave as agonists in bone cells leading to increase bone mineral density and quality. They inhibit bone resorption by increasing osteoclast apoptosis and in a lower capacity SERMs can increase osteoblast activity. However, because the main action is via inhibiting osteoclast activity they are considered as bone resorptive drugs without having the side effects, such as osteonecrosis of the jaw (ONJ) observed in patients treated with bisphosphonates (such as alendronate) [84].

## 4. Discussion and Future Directions

Despite the exponential increase in reports on the study of estrogen signaling over the past twenty years, the precise mechanisms of action in exerting the different effects in the musculoskeletal system are still to be determined. The ability of estrogen to signal not only through its nuclear receptors, ERα and ERβ, but also through membrane-bound receptors, e.g., GPER1/GP30, the possible crosstalk with other signaling pathways, e.g., BMP4 signaling pathway, epigenetic regulation of estrogen receptor itself, and recruitment of coactivators that are able to modify DNA methylation and histone arrangement might shed a light on explaining the wide effects of estrogen in bone. Additionally, new evidence is emerging that ERs can be activated without ligand stimulation. While this newly discovered mechanism of action is becoming a hot topic of study in the cancer field, there are not many reports in the bone field regarding this new ER signaling. In 2014, Takai et al. reported that unliganded ERα stimulates bone sialoprotein (BSP) a tissue-specific protein expressed by osteoblasts, odontoblasts, and cementoblasts during mineralization [18].

Estrogen depletion due to menopause or hysterectomy, or due to genetic disruption in its receptors, is detrimental to the whole body resulting in several physical and psychological changes. The systemic impact of estrogen depletion makes it impossible to understand the effect of estrogen in one specific organ due to the potential effects of its depletion in other endocrine organs. Therefore, one question that still needs to be answered is does impairment of estrogen signaling in one specific organ, like muscle or brain, affects other tissues such as bone? Answers to these questions can be obtained using the LoxP/Cre system. Several original articles have been published using animal models developed with this approach. However, most studies focus on deleting one of the receptors, ERα in most studies, in a specific bone cell and observing the effects in the skeletal tissue itself. However, until now very few reports focus on analyzing the effects of deletion of estrogen receptors in bone and the effects on other organs. Another area of high importance to fully understand the role of estrogen in the musculoskeletal system is the analysis of both genders in the same study. It is well accepted that estrogen plays key roles in both female and male health. However, some studies pursuing to understand the role of estrogen focus on the female group alone, missing important observations in the males. Furthermore, these studies must be done in the context of aging and especially in the context of sudden estrogen depletion such as menopause or hysterectomy. The results from these important studies can greatly help in the prevention, treatment, and management of age-related musculoskeletal diseases such as osteoporosis and sarcopenia. More reproducible, comprehensive, and conclusive experiments are needed to have a clear understanding of the specific role and the precise mechanism of estrogen in the musculoskeletal system and its crosstalk with other organs.
